# Correction to Covalent Triazine Frameworks Embedded with Ir Complexes for Enhanced Photocatalytic Hydrogen Evolution

**DOI:** 10.1021/acsaem.2c02271

**Published:** 2022-08-11

**Authors:** Nanfeng Xu, Yingxue Diao, Zhengtao Xu, Hanzhong Ke, Xunjin Zhu

Near the time of the submission of our now published manuscript^1^ an article on a closely related subject appeared^2^ that we were unaware of. Had we been aware of this important prior work, we certainly would have referenced it. For this reason we are now publishing this minor correction so the prior article is properly cited and the two articles in the record (and on indexing services) are more transparent to the community.
